# MicroRNA Regulation of Telomerase Reverse Transcriptase (TERT): Micro Machines Pull Strings of Papier-Mâché Puppets

**DOI:** 10.3390/ijms19041051

**Published:** 2018-04-01

**Authors:** Ammad Ahmad Farooqi, Qaisar Mansoor, Nada Alaaeddine, Baojun Xu

**Affiliations:** 1 Institute of Biomedical and Genetic Engineering (IBGE), Islamabad 44000, Pakistan; ammadfarooqi@rlmclahore.com (A.A.F.); qmibge@gmail.com (Q.M.); 2 Regenerative Medicine Laboratory, Faculty of Medicine, Saint-Joseph University, Beirut 1107-2180, Lebanon; nada.aladdin@usj.edu.lb; 3 Food Science and Technology Program, Beijing Normal University-Hong Kong Baptist University United International College, Zhuhai 519087, China

**Keywords:** telomere, telomerase, microRNA, TERT, cancers

## Abstract

Substantial fraction of high-quality information is continuously being added into the existing pool of knowledge related to the biology of telomeres. Based on the insights gleaned from decades of research, it is clear that chromosomal stability needs a highly controlled and dynamic balance of DNA gain and loss in each terminal tract of telomeric repeats. Telomeres are formed by tandem repeats of TTAGGG sequences, which are gradually lost with each round of division of the cells. Targeted inhibition of telomerase to effectively induce apoptosis in cancer cells has attracted tremendous attention and overwhelmingly increasingly list of telomerase inhibitors truthfully advocates pharmacological significance of telomerase. Telomerase reverse transcriptase (TERT) is a multi-talented and catalytically active component of the telomerase-associated protein machinery. Different proteins of telomerase-associated machinery work in a synchronized and orchestrated manner to ensure proper maintenance of telomeric length of chromosomes. Rapidly emerging scientific findings about regulation of TERT by microRNAs has revolutionized our understanding related to the biology of telomeres and telomerase. In this review, we have comprehensively discussed how different miRNAs regulate TERT in different cancers. Use of miRNA-based therapeutics against TERT in different cancers needs detailed research in preclinical models for effective translation of laboratory findings to clinically effective therapeutics.

## 1. Introduction

Cancer is therapeutically challenging and molecular biologists have provided near to complete resolution of the signaling landscape of oncogenic and tumor suppressor pathways. Telomerase reverse transcriptase (TERT) is a specialized enzyme that is distinguishable from other reverse transcriptases by a unique mode of action that promotes template re-alignment to enable continued synthesis of multiple DNA repeats. TERT synthesizes multiple DNA repeats without dissociation from the telomere. Studies have shown that telomerase is structurally composed of a reverse transcriptase (TERT), which uses an RNA component (TERC) to dock onto the 3′ single-stranded telomeric end. TERT then processively synthesized telomeric repeats from template provided by TERC, before it got dissociated from attachment site. All telomerase RNAs possessed a 3′ end element which played central role in enhancing its stability. In TERC, there are two stem-loop structures separated by an H-box (ANANNA) and ACA motif (H/ACA). Binding of telomerase factors dyskerin, NHP2 and NOP10 at the H/ACA motif formed a ‘pre-ribonucleoprotein complex’, before formation of a mature RNP. TERC structurally interacted with chaperone Telomerase Cajal Body Protein-1 (TCAB1), which catapulted it to the Cajal bodies. Recruitment to the telomeres in S-phase is mediated by the protective complex shelterin. Highly organized assembly of the telomerase complex synchronously with functionally active co-factors for stability, maturation and subcellular localization was noted to be essential for its biological functionality and thus telomeric maintenance [1,2,3].

Most cancer cells strategically activated telomerase for proper telomeric maintenance to fuel replicative ability [4]. In cancers, rate-limiting factor for telomerase activity is TERT expression. Telomerase-positive cultured human cells contained ~500 TERT molecules and ~1150 TERC molecules per cell. Estimated number of functionally active and molecularly assembled telomerase complexes ranged from 20 to 240 complexes/cell [4]. Findings pinpointed towards a pool of unassembled components of telomerase. However, these components had the ability to assemble into functionally active machinery as per needs of the cell. Specific activity of telomerase and of overexpressed super-telomerase had previously been calculated. Interestingly, ~60 nucleotides were calculated to be incorporated per telomerase/minute, with *K*_m_(dGTP)-17 μM [4]. Thematically, term ‘super-telomerase’ was used for the cells which demonstrated massive telomerase activity and had by co-overexpression of TERC and TERT [5]. In super-telomerase HT1080 cells, 40 times increase in telomerase activity was noted along with significant lengthening of telomeres from 2.5 to 20 kb [5]. Transcriptional regulation of TERT is tightly controlled and pieces of evidence pinpoint towards association of telomerase activity with TERT expression [6]. Ectopic expression of an epitope-tagged TERT in telomerase-negative cells induced telomerase activity which was comparable to the activity observed in immortalized telomerase-positive cells [6].

**Figure 1:**
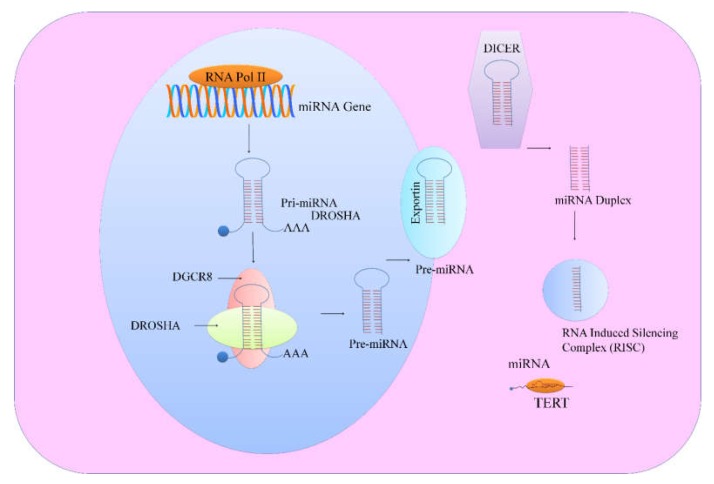
Schematically represents miRNA biogenesis and different steps involved in its maturation. DICER, one RNase III enzyme; DROSHA, another RNase III enzyme; TERT, telomerase reverse transcriptase.

Mechanisms associated with microRNA biogenesis and functions have gained tremendous appreciation and it is now more interpretable that majority of miRNA genes are transcribed by RNA polymerase II, transcription factors and epigenetic regulators in the nucleus [7,8]. miRNAs are transcribed from introns of genes which encode proteins, while various other miRNAs are transcribed from miRNA gene loci. Length of pre-miRNA is over 1kb and either produces a single miRNA or contains cluster of two or more miRNAs which are transcribed from the same primary transcript. Pre-miRNAs are processed by cellular machinery, which comprises DROSHA (RNase III enzyme) and its functionally efficient partner, DiGeorge syndrome critical region 8 (DGCR8). Recent studies have helped us to gain insights into the structure of DROSHA and its functionality has been extensively elucidated. DROSHA contained two RNase III domains and cropped stem–loop to release a hairpin-shaped RNA of ~65 nucleotides (pre-miRNA). Microprocessor precisely cleaved dsRNA ~11 bp from the junction with the flanking single-stranded-RNA to generate hairpin-shaped pre-miRNAs which had overhangs at the 3′ end of either 1 nucleotide or 2 nucleotides. Pre-miRNAs were shipped to the cytoplasm by exportins for further processing by DICER, an RNase III enzyme. DICER binding to the end of the pre-miRNA was necessary for critical positioning of its catalytically active RNase III domains (Figure 1). These catalytically active RNase III domains asymmetrically cleaved double stranded-RNA stem. DICER mediated processing resulted in the formation of mature ~22 nucleotide miRNA duplexes which had 2 nucleotide 3′ overhangs. TRBP played important role in promoting structural association of DICER1 with the Argonaute proteins to participate in the assembly of the RNA induced silencing complex (RISC) (Figure 1). Emerging research work has emphasized on the selective loading of the strand. In accordance with this concept, ‘passenger’ strand was discarded and the mature ‘guide’ miRNA strand remained bound to one of the Argonaute proteins. Guide RNA is intricately channelized through characteristically unique domains of Argonaute protein to reach the PAZ domain that binds uniquely to the 3′ end of the guide RNA [9].

There has been an exponential rise in the high-quality research related to most recent strategies to target telomerase in different cancers. miRNA regulation of TERT has paradigmatically shifted our understanding and leveraged molecular oncology to another level of sophistication and diversity, leading to many more exciting discoveries. In this review we have comprehensively summarized miRNA regulation of TERT in different cancers. We set spotlight on the mechanisms by which miRNAs regulate TERT. Certain clues have emerged which have compelled researchers to focus on TERT mediated regulation of different miRNAs. This emerging theme will also be discussed in later part of the review. We will also provide an overview of the competition between miRNAs to regulate TERT.

## 2. MicroRNA Regulation of TERT

TERT was found to be over-expressed in different cancers [10,11]. Interestingly, TERT worked synchronously with different regulators and transcriptionally controlled expression of target genes [12]. Given the wealth of knowledge unfolding extra-telomeric roles of TERT in different cancers, this review confines itself to the theme elucidating miRNA regulation of TERT in various cancers. List of miRNAs involved in TERT regulation is presented in Table 1.

**Figure 2:**
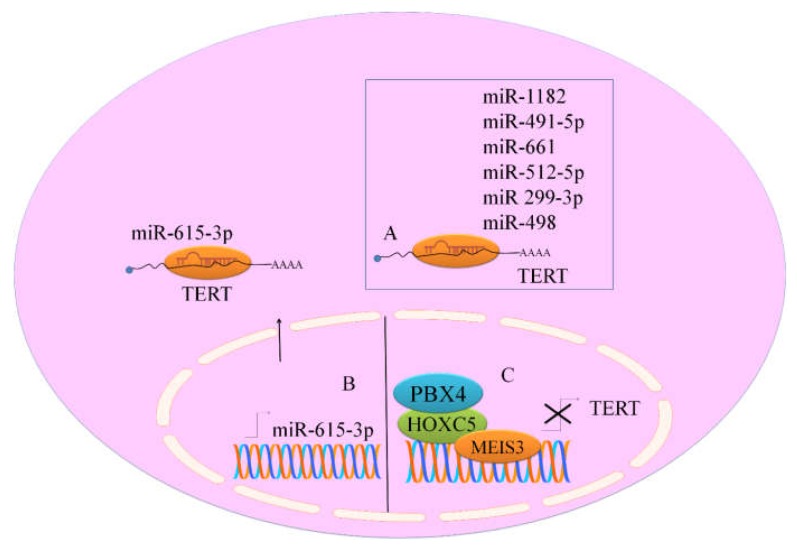
Regulation of telomerase by different miRNAs (**A**). miR-615-3p (**B**) is transcribed from *HOXC5* (**C**). Both *HOXC5* and miR-615-3p negatively regulated TERT. *HOXC5* promoted loading of PBX4 and MEIS3 to repress TERT. *HOXC5*, one of several homeobox HOXC genes located in a cluster on chromosome 12; PBX4, pre-B-cell leukemia transcription factor 4; MEIS3, Meis homeobox-3.

hTERT 3′UTR (560 bp) and 5′UTR (58 bp) were cloned combinatorially or individually at specific ends of Renilla luciferase reporter in psiCHECK2 [20]. hTERT 3′UTR was a central player in regulation of hTERT differential expression in various cell lines. It was noted that miR-615-3p quantitatively controlled hTERT in cancer cells. Various findings provided evidence of presence of miR-615 in intron-1 of *HOXC5* and therefore transcriptional activation of *HOXC5* also triggered miR-615 expression (Figure 2). *HOXC5* over-expression resulted in significantly reduced hTERT, low telomerase activity, and sequential reduction of telomeres in different cancer cells. Mutational inactivation of the homeodomain, significantly compromised *HOXC5*-regulated hTERT suppression and telomerase inactivation in both PC-3 cells and HeLa cells [20]. *HOXC5* binding site was identified −20 kb upstream of transcription start site of hTERT in PC-3 cells (Figure 2). HOXC5 repressed TERT by interfering with the long-range interactions between TERT promoter and its distal enhancers. HOXC5 promoted loading of Pre-B-cell leukemia transcription factor 4 (PBX4) and Meis Homeobox-3 (MEIS3) to repress TERT (Figure 2). TERT mRNA levels were drastically reduced in cancer cells which transiently overexpressed PBX4 or MEIS3 combinatorially with HOXC5 to transcriptionally repress TERT [20]. PC-3 cells which individualistically expressed HOXC5 or miR-615-3p, or co-expressed miR-615-3p and HOXC5 were inoculated subcutaneously into immuno-compromised mice for detailed analysis of tumor growth. Data clearly suggested that tumors derived from *HOXC5* over-expressing or miR-615-3p and *HOXC5* over-expressing PC-3 cells were significantly smaller. *HOXC5* over-expression in PC-3 resulted in shortened telomeres and formation of telomeric-dysfunction induced foci (TIFs) [20]. It seems clear that miR-615-3p is present within intron of *HOXC5* gene and co-transcribed with *HOXC5*. Therefore, whenever, *HOXC5* gets transcribed, miR-615-3p will also be transcribed. *HOXC5* and miR-615-3p worked harmoniously for transcriptional and post-transcriptional repression of TERT respectively.

miR-532 and miR-3064 combinatorially targeted hTERT in ovarian cancer [21]. Ovarian cancer cells which ectopically expressed miR-532/miR-3064 had markedly reduced proliferation and invasion potential. However, miRNA-532 or miRNA-3064 inhibition stimulated proliferation and invasion of ovarian cancer cells. Tumor growth was significantly reduced in mice xenografted with miRNA-532 or miRNA-3064 over-expressing ES-2 cells [21].

miRNA-296-5p and miRNA-512-5p are reportedly involved in targeting of hTERT in MDA-MB-231 breast cancer cells [22]. However, epigenetic inactivation of these miRNAs saved hTERT from miRNA-mediated inhibition. 5-aza-2′-deoxycytidine, a DNMT inhibitor efficiently reduced DNA methylation levels of CpG rich regions and induced upregulation of miRNA-296-5p (5 fold) or miRNA-512-5p (70 fold) in MDA-MB-231 cells. Reduction in TERT levels and telomeric shortening were observed in the cells which had high expression of mature miR-296-5p or miR-512-5p. Vorinostat also known as suberanilohydroxamic acid (SAHA) is a member of family of histone deacetylase (HDAC) inhibitors. SAHA and 5-aza-2′-deoxycytidine synergistically induced a 90-fold increase in miRNA-512-5p as compared to a 5-aza-2′-deoxycytidine induced 25-fold increase in miRNA-512-5p in MDA-MB-231 cells [22].

Increasingly it is being realized that alternative lengthening of telomeres (ALT) and telomerase activity (TA) are the mechanisms opted by cellular machinery for the maintenance of telomeres [23]. miR-380-5p was considerably down-regulated in TA-positive diffuse malignant peritoneal mesothelioma (DMPM). Ectopically expressed mature miR-380-5p reduced growth of DMPM cells but the growth was not inhibited in telomerase-negative or normal mesothelial cells. miR-380-5p exerted inhibitory effects on growth of DMPM cells mainly through inhibition of TA [23]. Furthermore, miRNA-380-5p-mediated inhibitory effects on TA were paralleled by a notable reduction in Telomerase-associated protein-1 (TEP1), which played central role in proper functioning of the holoenzyme. Mechanistically it was revealed that miRNA-380-5p interacted with open reading frames of TEP1 and 3′-UTR of Testis-specific protein, Y-encoded-like 5 (TSPYL5) gene. More importantly, miR-380-5p time-dependently reduced growth of the cells, which was associated with remarkably reduced levels of TEP1 and TSPYL5. After 3 months of weekly repeated miR-380-5p transfections, STO cells demonstrated an increase in mature miR-380-5p which was associated with remarkably reduced TEP1 mRNA and TA inhibition [23]. Slightly increased mean telomeric lengths (about +1 Kb) were noted in cells consistently reconstituted with miR-380-5p after 3 months. Moreover, long-term miRNA-380-5p transfectants revealed significantly reduced ATRX and detectable level of C-circle DNA [23].

Data clearly suggested that miR-380-5p interfered with telomerase activity and reduced growth of the cells and induced apoptosis in specific DMPM models. However, long-term ectopically reconstituted cells showed characteristically unique features which were reminiscent of an ALT phenotype (‘ALT-like’) that provided DMPM cells with compensatory mechanisms to fuel their growth even when TA was partially inhibited. In the upcoming section we summarize how tumor suppressor miRNAs negatively regulate TERT.

## 3. miRNA Mediated Negative Regulation of TERT

It has been persuasively documented that TERT over-expression played central role in cancer development and progression. However, circumstantial studies have also highlighted miRNA regulation of TERT in different cancers.

14-3-3 Protein isoforms θ and ζ effectively inhibited binding of CRM1/exportin-1 to TERT nuclear export signal (NES) motif [24]. Structurally, these isoforms shared a conserved terminally located domain at carboxyl end that interacted with TERT and enhanced its accumulation in the nucleus. Interestingly, 14-3-3ζ was directly controlled by miR-375. mRNA and protein levels of 14-3-3ζ were reduced upto ~50% in HeLa cells transfected with miR-375 mimics [24]. Data clearly suggested that miR-375-regulated targeting of 14-3-3ζ considerably reduced accumulation of TERT in the nucleus.

Bioinformatics and dual-luciferase assays suggested that miR-1182 regulated TERT by binding to its open reading frame. Furthermore, miR-1182 recognized elements in the region which spanned between 2695 and 2719 of TERT mRNA [25]. Tumor growth and metastasizing potential of SGC-7901 cells which stably expressed miR-1182 was significantly lower in xenografted mice [25].

## 4. miR-34a Regulation of Foxm1/C-Myc Signaling Cascade to Repress TERT

Certain hints have emerged which highlighted that miRNA effectively inhibited positive regulators of TERT in different cancers [26]. miR-34a efficiently targeted Forkhead Box M1(FOXM1) and c-Myc in hepatocellular carcinoma. Previous studies revealed that FoxM1 transactivated c-Myc promoter mainly through both its P1 and P2 TATA box. c-Myc over-expression induced an increase in TERT in FOXM1 silenced SMMC-7721 cancer cells [26]. It was concluded that miR-34a inhibited telomerase significantly, mechanistically through FoxM1/c-Myc signaling cascade.

## 5. miRNA Mediated Positive Regulation of TERT

Certain clues have emerged which highlighted TERT regulation through PTEN inhibition and ERK1/2 pathway activation [27]. Detailed analysis demystified that miR-21 negatively regulated PTEN to promote ERK1/2 pathway mediated activation of TERT [27]. Paired-like homeodomain1 (PITX1) transcriptionally repressed TERT in melanoma cells [28,29]. miR-19b indirectly promoted TERT expression by negative regulation of PITX1 [29]. Max dimerization protein 1 (Mxd1) is directly inhibited by miR-202 [30]. Detailed mechanistic insights provided new information about binding of Mxd1 to the promoter region of TERT which substantially reduced c-Myc binding to TERT promoter in pancreatic cancer cells. Furthermore, TERT expression was reduced in MiaPaCa-2 and PANC-1 cells exposed to 3-Cl-AHPC(E)-4-[3-(1-adamantyl)-4-hydroxyphenyl]-3-chlorocinnamic acid [30]. Findings clearly suggested that miR-202 targeted Mxd1 to increase TERT expression in cancer cells.

### 5.1. miR-103 Induced an Increase in Level of TERT through Targeting of AKAP12

AKAP12 (A kinase anchor protein 12), a family member of A-kinase scaffold proteins played contributory role in cancer suppression [31]. Tumor forming capacity of HepG2 cells which ectopically expressed miR-103 was notably higher. While tumors formed by HepG2 cells which ectopically expressed AKAP12 were smaller in size. AKAP12 knockdown significantly increased PKCα activity and enforced expression of AKAP12 significantly reduced PKCα activity. AKAP12 over-expression resulted in suppression of phosphorylated TERT, reduction in nuclear accumulation of TERT and markedly reduced activity of telomerase [31]. On the contrary, AKAP12 knockdown increased phosphorylation of TERT and promoted accumulation of TERT in the nucleus. miR-103 over-expression significantly increased PKC activity levels, increased levels of TERT and increased nuclear accumulation of TERT [31]. AKAP12 mRNA was targeted by miR-103. AKAP12 was noted to inhibit protein kinase C-α (PKCα). PKCα phosphorylated TERT and promoted its nuclear accumulation to transcriptionally regulate different genes. These clues are opening new horizons for investigation of extra-telomeric roles of TERT in different cancers.

### 5.2. miR-138

TERT is directly controlled by miR-138 in different cancers. miR-138 inhibited TERT mRNA. Target specificity was found between miR-138 and the TERT 3′UTR as evidenced by a luciferase reporter assay. Growth of the tumor was notably reduced in mice xenografted with miR-138 over-expressing HeLa cancer cells [32]. Hsa_circ_0020397, an exon-derived circRNA originated from the DOCK1 and acted as a sponge for different miRNAs [33]. Linear hsa_circ_0020397 cloned into a psiCHECK-2 vector was co-transfected with miR-138 mimics into HCT116 cells. Findings obtained from real-time PCR clearly indicated that when hsa_circ_0020397 over-expression vector was transfected into cells, hsa_circ_0020397 did not alter miR-138 expression but significantly up-regulated TERT [33]. Data clearly suggested that hsa_circ_0020397 did not alter miR-138 expression but inhibited the miR-138-regulated targeting of different genes.

It seems clear that tumor suppressor miRNAs are strategically inhibited to enhance TERT expression. We still have unanswered questions which need detailed research. Apart from circular RNA mediated repression of tumor suppressor miRNAs, long non-coding RNAs also serve as versatile sponges to sequester tumor suppressor miRNAs from target mRNAs.

### 5.3. Long Non-Coding RNA (BC032469) Served as a Sponge for TERT Targeting miR-1207-5p

**Figure 3:**
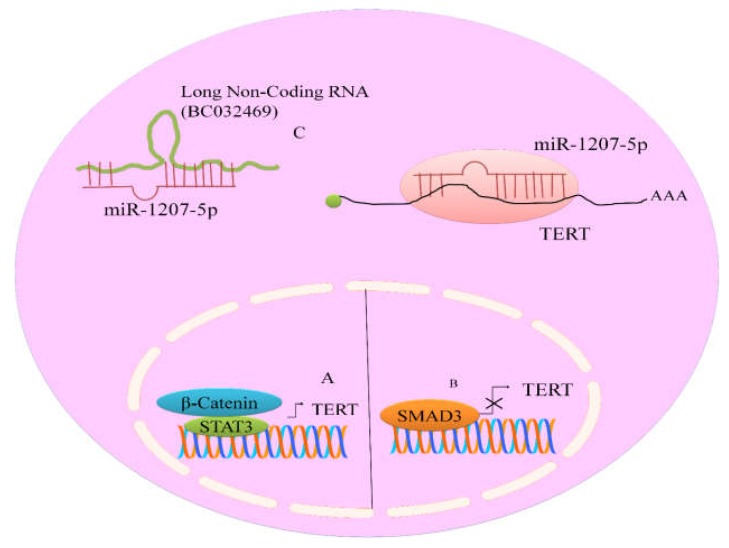
(**A**) Positive regulation of TERT by WNT signaling pathway specific protein (β-catenin) and STAT3; (**B**) SMAD transcriptionally repressed TERT; (**C**) BC032469, a lncRNA contained sequences which showed complementarity to miRNA-1207-5p seed regions.

BC032469, a lncRNA was noted to be frequently over-expressed in gastric cancer [34]. It has recently been convincingly revealed that BC032469 contained sequences which showed complementarity to the miR-1266 and miRNA-1207-5p seed regions (Figure 3). RNA interference technique against BC032469 resulted in up-regulation of miR-1207-5p and miR-1266. More specifically, up-regulation of miR-1207-5p was observed in BC032469-silenced SGC-7901 cells. BC032469 acted as a competitive endogenous RNA and structurally associated with miR-1207-5p to inhibit it. Therefore, this sponging mechanism inhibited miR-1207-5p mediated targeting of TERT [34].

### 5.4. TERT Mediated Repressive Effects on miR-29a

In this section, we draw attention to the recent progress made in outlining the mechanisms opted by TERT to repress expression of tumor suppressor miRNAs. Integrin β1 (ITGB1) was significantly increased in TERT over-expressing SGC7901 cancer cells [35]. ITGB1 was quantitatively controlled by miR-29a in cancer cells and astonishingly, TERT negatively regulated miR-29a expression. To check whether TERT could modulate miRNA-29a, BGC-823 and SGC-7901 cells were co-transfected with miR-29a and TERT [35]. TERT knockdown resulted in an increase in the miR-29a expression. Tumor growth was remarkably reduced in mice xenografted with miR-29a over-expressing SGC-7901 cells. Moreover, smaller and fewer metastases were noted in the livers and lungs of mice injected with miR-29a over-expressing SGC-7901 cells [35].

## 6. Push and Pull between miRNAs to Regulate TERT

miRNA-346 promoted growth of HeLa cancer cells, whereas inhibition of miRNA-346 significantly reduced growth potential of cervical cancer cells [36]. Bioinformatics tools provided information about presence of miR-346 binding region in 3′UTR of TERT. A reporter assay was used for experimental verification of binding of miR-346 directly to 3′UTR of TERT [36]. TERT 3′UTR segment which contained either the binding site for miR-346 or a mutated sequence was inserted downstream to the reporter gene [36]. pri-miR-346 increased fluorescence of reporter, while ASO-miR-346 significantly suppressed the reported fluorescence. Data clearly suggested that more than 2 fold increases were noted in TERT (mRNA and protein) in HeLa cells transfected with pri-miR-346; however antisense oligonucleotides against miR-346 reduced it by 45%. Similarly, telomeric repeat amplification protocol assays demonstrated that pri-miRNA-346 substantially increased, but ASO-miR-346 exerted repressive effects on telomerase activity [36]. Furthermore, actinomycin D was used to block the synthesis of new transcripts for analysis of TERT mRNA stability in HeLa cells. Additionally, pri-miRNA-346 increased, while ASO-miR-346 destabilized TERT mRNA in cells treated with actinomycin D. It was surprising to note that miR-138 and miR-346 shared same binding site in TERT 3′UTR for regulation of its expression. Reporter vector which contained TERT 3′UTR segment equipped with binding sites for miR-138 and miR-346 was transfected along with varying concentrations of pri-miRNA-138 or pri-miRNA-346 in HeLa cells [36]. Expression levels of the reporter gene and the ratios of pri-miR-346/pri-miR-138 correlated positively when fixed concentrations of miR-346 or miR-138 were used. Dot blot hybridization technique was used to investigate whether or not miR-346 or miR-138 competed with each other for binding to mRNA of TERT. In this technique, ^32^P-labelled miRNAs (hot-miR-138, hot miR-346) were used as probes [36]. Furthermore, as a negative control, non-labelled miR-16 (cold miR-16) was used to analyze competitive binding of miR-138 and miR-346 to TERT 3′UTR. More importantly, if the level of cold miR-138 was increased, it exerted inhibitory effect on the binding of hot miR-346 to 3′UTR segment of TERT [36]. Since Argonaute (AGO2) was the central effector of RNA-induced silencing complex (RISC), it was inhibited using RNA interference strategy to see if AGO2 played essential role in miR-138- and miR-346-directed modulation of TERT. Knockdown of AGO2 resulted in miR-138-directed down-regulation of TERT mRNA and protein [36].

RNA immunoprecipitation assays indicated significant association of miR-138 and TERT mRNA to the AGO2 protein- containing complex, but miR-346 was bound to AGO2 complex in low fraction. miR-346 over-expression reduced association of TERT mRNA with AGO2, because of increased binding of TERT mRNA to miR-346. AGO2 depletion resulted in the reduction of miR-138 and TERT (mRNA) loading to AGO2 protein-containing complex and there was an increase in the loading of TERT (mRNA) and miRNA-346 to GRSF1 complex. GRSF1 belongs to the family of hnRNP F/H RNA binding proteins and involved in modulation of miR-346-directed TERT up-regulation by binding to middle sequence motif (CCGCAU) present in miR-346 which consequently formed a ‘bulge loop’. This mechanism facilitated the loading of TERT mRNA to ribosomal machinery to initialize translation. Excitingly, miRNA tactfully promoted TERT translation in an AGO2-independent manner [36]. Data clearly suggested that ‘bulge loop’ of miRNA-346 (CCGCAU) was critical in TERT up-regulation.

GRSF1 over-expression increased TERT, while GRSF1 knockdown exerted repressive effects on its expression. There was approximately 60% reduction in TERT protein in GRSF1 silenced cells transfected with miRNA-346. miRNA-346/TERT 3′UTR fragment structurally associated with GRSF1 complex through miRNA-346 ‘CCGCAU’ motifs to promote TERT expression [36].

## 7. Interplay of TERT, miRNAs and Signaling Pathways: More Questions than Answers

One largely unheralded theme of this story is the extent to which intracellular signaling cascades regulate expression of TERT via positive or negative regulation of miRNAs and how miRNAs regulate signal transducers of different pathways to modulate TERT expression.

Aberrantly active β-catenin transcriptionally up-regulated TERT expression in the epithelium of the small intestine in animal models [37]. Mechanistically it was shown that β-catenin recruited histone methyltransferases (HMTs) to trigger expression of TERT (Hoffmeyer). Luciferase reporter assays provided evidence that let-7g, miRNA-133a, miRNA-138, miRNA-342, miRNA-491, and miRNA-541 functionally interacted with the 3′UTR of TERT [35]. Furthermore, miR-342, miR-541 and miR-491 significantly regulated 3′UTR of Wnt pathway regulating genes (*TCF7*, *PAX5* and *MSI1*) [38]. These findings clearly suggested that targeted inhibition of Wnt pathway and TERT was necessary to inhibit cancer progression. Moreover, this report also gave us a clue that different miRNAs had the ability to simultaneously target transducer proteins of Wnt pathway and TERT.

Interestingly, as previously discussed about tumor suppressive effects exerted by different miRNAs, oncogenic miRNAs stimulated the expression of TERT by promoting the expression and function of JAK-STAT pathway. Consensus STAT3-binding sites (TTCNNNGAA) have previously been identified in the TERT promoter at 3308/3316 bp [39] (Figure 3). Moreover, miR-21, an oncogenic miRNA significantly promoted STAT3 expression and phosphorylation in LN229 and U87 cells. Use of antisense oligonucleotides against miR-21 markedly reduced STAT3 and TERT in treated cancer cells [39]. These findings will be helpful in the analysis of the pathways which stimulate TERT expression and how different miRNAs effectively promote signaling pathway-mediated increase in TERT expression.

However, different pathways negatively regulated expression of TERT in different cancers. TGFβ repressed the expression of TERT via SMAD3 (Figure 3). Inhibition of SMAD3 substantially impaired TGFβ mediated inhibitory effects on TERT [40].

There is sufficient experimental evidence related to miRNA mediated regulation of TERT but we still have incomplete information related to central role of 3′UTR of mRNA of TERT [41]. Longer 3′UTRs had greater number of microRNAs response elements (MREs) and alterations in the sizes of 3′UTRs had noteworthy impact on key biological mechanisms that operated through weakening or strengthening of the repressive effects of miRNAs [41]. It had previously been convincingly revealed that cancer cell lines had significantly higher levels of mRNA isoforms which had shorter 3′UTRs [42]. Shorter mRNA isoforms exhibited greater stability and produced ten-fold more protein, mainly through the loss of miRNA-induced repression. miRNA complementary sites were mutated and expression levels of different genes were studied. Six sites for miR-103/107 were mutated in the *DICER1* 3′UTR and three sites for miR-15/16 were mutated in the *Cyclin D2* 3′UTR. Luciferase activity was examined and data clearly suggested that loss of these miRNA sites induced up-regulation of these genes [42]. Future studies must converge on unraveling the effects of changes in 3′UTR of TERT and how it influenced miRNA mediated targeting of TERT in different cancers.

## 8. Conclusions

It will not be wrong if we say that the basics of the miRNA regulation of TERT have been demystified, however, we still have many unresolved outstanding questions. TERT has been noted to be enzymatically active in different cancers. TERT activation in different cancers has fuelled extensive research, producing sufficient knowledge about structure and functions of telomerase-associated machinery. However, it seems surprising to note that in spite of our rapidly evolving concepts about the linchpin role of telomerase and its associated machinery in cancer development and progression, only one telomerase inhibitor, imetelstat (GRN163L), has passed different preclinical trials to make its entry into different phases of clinical trials. miRNA regulation of TERT has added new layers of information to an already complex role of TERT in different cancers.

It is becoming sequentially more understandable that based on the positive and negative regulation of TERT, miRNAs are categorized into oncogenic and tumor suppressor miRNAs. Scientists have started to identify the miRNAs which cell-type-specifically control TERT but we still have insufficient understanding about the mechanisms used by TERT to transcriptionally inhibit tumor suppressors and stimulate oncogenic miRNAs. Future studies must converge on the identification of natural products and synthetic compounds which can inhibit TERT via stimulating the expression of tumor suppressor miRNAs. It will be also exciting to see how different tumor suppressor miRNA mimics can be used with natural products or telomerase inhibitors to synergistically inhibit cell proliferation and growth of the tumors in xenografted mice.

## Figures and Tables

**Table 1:** miRNAs involved in TERT regulation in different cancers.

TERT Targeting miRNAs	Cancer	References
miR-1182	Bladder cancer	[13]
miR-491-5p	Cervical cancer	[14]
miR-661	Glioma cells	[15]
miR-512-5p	Head and neck squamous cell carcinoma	[16]
miR-299-3p	Laryngeal cancer	[17]
miR-1207-5p, miR-1266	Gastric cancer	[18]
miR-498	Ovarian cancer	[19]

## Abbreviations

TERT	Telomerase reverse transcriptase
TERT	Telomerase activity
ALT	Alternative lengthening of telomeres
ALT	RNA induced silencing complex
